# Editorial

**Published:** 1873-01

**Authors:** 


### EDITORIAL.

### Annual Meeting of the Ohio State Dental Society.

The seventh annual convocation of this body was held in the city of Columbus, December 4th, 5th, and 6th.

There was a large number of the members present, and in addition to these a large number of visitors.

The meeting was a very good one; nearly all of the active members participating more or less in the proceedings.

Some excellent papers were read; and the discussions upon the different subjects presented were interesting and instructive; and many had occasion to say that it was good for them to be there. Quite a number of new, interesting and useful instruments and appliances were presented for inspection. Progress in the profession bears along with it great and important changes, and improvements in all these things.

It is every year becoming more apparent that the science of the profession is making rapid development. It is very gratifying to have a consciousness that it cannot, with truth, much longer be said that "though the American dentists may exceed any others in a mechanical and artistic aspect, yet as to its science there are those of other countries very much our superiors."

Now upon this we will not stop to discuss, but simply press on in the work before us.

The present meeting is an additional illustration of the statement we have so often made, that our profession is making very rapid growth and development.

Many regrets were expressed for the absence of some valued members who have usually been present, and added largely to the interest of the meetings. Of these we will mention Watt, Spellman, Williams, Butler and Berry; there are a number of others equally worthy of mention; indeed there were enough absentees, who of themselves would have constituted a grand meeting. We trust that at the next annual meeting they will with one accord be in the midst of their brethren.

### Ohio State Dental Board.

The State Board of Dental Examiners held its annual meeting in the city of Columbus, on Teusday, December 3rd, and held sessions during four days. Between eighty and ninety dentists appeared before the Board for examination. The majority of whom passed the ordeal very satisfactorily indeed. In many cases it was plainly evident that considerable effort had been expended in preparation for the occasion; in some instances the preparation was of such a character as to be decidedly advantageous; in the examination in other cases it missed the point altogether.

All such study and preparation will be of permanent benefit. In other cases reliance was placed wholly upon what had been learned in early life, or at the time of studentship, together with what had been obtained by experience; the latter will be of value in proportion to the improvement made of it.

The ability of men to utilize experience, varies exceedingly; some will learn and observe much that will be wholly overlooked by others. And no where perhaps will such facts be more palpably brought out than in examinations like these.

The position of the members of this Board is by no means an enviable or desirable one. The responsibilities are great, and the labor onorous during the sessions, and the various annoyances to which the members are subjected, can hardly be appreciated by those who have not personal knowledge of the matter.

The names and residence of all those who have passed the Board since its last annual meeting will be given in the minutes of the State Society, which will be published in the February number of the REGISTER.

There will be a meeting of the Board for the examination of applicants in Cincinnati at the time of the meeting of the Mississippi Valley Dental Society, which will be about the 1st of March next. The precise time will be given in the REGISTER of February.

### Transactions of the American Dental Association.

The volume of transactions of the meetings of the American Dental Association for the years 1871 and 1872, is at hand. It makes a very neat volume. The Publication Committee have showed ability and good taste; as usual, however, a few typographical errors have crept in as will be seen by the corrections noted below, in one paper, viz: that of Dr. Sage, on Therapeutics. These and others occur doubtless because the proof was not sent to the writers. Publication Committees are sometimes placed in an unpleasant position by just such things.

The volume is one that no dentist should be without, for it contains much that is valuable. It may be obtained of Dr. M. S. Dean, the Chairman of the Publication Committee, Chicago, or of the Treasurer, Dr. W. H. Goddard, Louisville, Ky.

### Corrections.

In the published transactions of the American Dental Association for 1872, the report on Dental Therapeutics, contains some typographical errors which, it seems to the writer, quite important to correct. I will mention only the most prominent.

For "18½," on page 73, sixth line from top, read "1872;" for "if the sensitiveness might be due," on page 74, third line from bottom, read "if the sensitiveness was due;" for "maganese," on page 79, third line from top, read "manganese;" for "cymglic," on page 83, first line at bottom, read "cymylic;" for "Ajonan," on page 84, tenth line from top, read "Ajowan;" for "living membrane." on page 85, ninth line from bottom, read "lining membrane;" for "thymilic," on pape 85, nineteenth line from top, read "thymylic", for "Chloride of Lime," on page 86, seventeenth line from bottom, read "Chloride of Zinc;" for "instead," read "intact;" for "ulderised bone," read necrosed bone;" for "organic," on page 87, read "inorganic."

H. L. Sage.

### Ethics.

Frequently such advertisements as the following are sent to us with a request for an opinion as to the propriety of such announcements. The following just received:

**SCIENTIFIC DISCOVERY.**

**TEETH EXTRACTED.**

*POSITIVELY PAINLESS.*

Without the use of nitrous gas, chloroform, or freezing process.

Artificial Teeth inserted without plate or clasps. Plumpers adjusted to sets, to set out the cheeks and restore youthful appearance. Weighted lower sets.

☞Persons coming to the city can have teeth inserted the same day.

**Dr. Q. Blowhardt,**

[LATE OF NEW YORK],

GRADUATE DENTIST,

Room 1090, No. 74016 W. Street.

Now as to the propriety of resorting to this kind of advertisement, there is no diversity of opinion in the minds of truly professional men. It, to say the least, savors strongly of quackery. The employment of such means may generally be regarded as evidence of a want of proper ability. It is employed to supplement that which is far more desirable.

The dentist who is conscious of his professional strength and ability, is always disposed to rely upon these as the great elements of his success, and not upon the means used by charlatans and quacks everywhere, viz: by pretentious, self-laudatory, and untruthful advertisements, which deceive the unwary, and disgust the intelligent and discriminating.

In all intelligent communities such advertisements fall short o the point aimed at; and instead of securing that which is desirable, in a business aspect, it seems only to draw about one a class of persons whose contact, in a business way, so far from proving a stimulous is depressing and demoralizing; and so it is found in almost all instances that those fullsome advertisers either never possessed any desirable professional ability, or if they did, they came to a deliberate conclusion to prostitute their chosen profession to a very degraded level. Generally these advertisemants contain either directly or by implication that which is not true, and so far as belief is given to them are they a means of deception. " But if we may not advertise, how shall we obtain business?"

To this we will endeavor to give an anwer ere long.

### Gutta Percha.

We have just received the following in reference to vulcanizing gutta percha for dental plates from Dr. W. E. Driscoll, which accords very nearly with the results obtained by others. Indeed similar results were obtained with gutta percha fifteen years ago, but it was abandoned becanse of a little more difficulty in manipulating it.

"Agreeably to your request to let you know when I succeed satisfactorily with vulcanized gutta percha, I enclose a specimen which I think settles the question.

It is vulcanized too hard for a practical plate, but I did that when vulcanizing a rubber one at the same heating.

The only difference in the manipulation from that of rubber is that about one third as much sand must be used as plaster in making the mould, and perfectly dried before packing. Then the mould or flask must not be more than just barely warm enough to make the gutta percha press into proper shape.

Thirty minutes is short enough time to bring the temperature up to three hundred and twenty degrees, and fifty-five minutes enough to keep it at that point.

I have a number of plates of this material that have been in use two months, and seem to be fully as good as rubber, and resembles rubber plates so much that no one can tell the difference by looking at them only.

This is a matter of so much interest to the greater portion of the profession, I think you will confer a favor upon them by calling attention to it in the REGISTER."

### A New Tongue Holder.

We have had in use for some time "Osborn's Tongue Holder and Duct Compressor," which accomplishes the object proposed by it better than any appliance hitherto in use. It is a very good appliance indeed, and one which every operator will find invaluable in many cases. A cut of it will be found in the advertiseing sheet of the REGISTER.

### Changes, Professional.

Dr. B. T. Spellman, recently of Warren, Ohio, has removed to Detroit, and formed a business connection with Dr. H. Benedict, an old established practitioner of that city. We congratulate the profession of Michigan upon the acquisition of Dr. Spellman to their ranks; but we in Ohio *cannot* afford to spare such men.

We now promise the profession in Michigan that we shall retaliate upon them at the first opportunity, which will possibly not be long delayed.

Also Dr. D. R. Jennings, formerly of Ravenna, is now in Cleveland, hewing his way right along in the front ranks, indeed he wouldn't be any where else. The Doctor has got into such a shape that this is about the only place into which he would fit.

### The Rubber Contest.

In reply to the numerous inquiries in reference to the common cause which the profession is making with the owners of the Cumming's patent, we would say that we have information that the Committee of the American Dental Association, as well as the Committee of the Ohio State Society are at work organizing for a concert of action in all the states to resist the demands of the Dental Vulcanite Company.

In the matter of the Gardner Appeal Case, S. S. White is pressing his issue upon them. And Bacon and Company are using all the ordinary and extraordinary means to postpone a hearing. It is not probable that it will be delayed long after the holidays. The purpose of this delay it would seem is to allow as many of the profession as possible to send in their annual contributions during the month of January. In the event Mr. White is successful; a good portion of this material aid would be withheld.

We would suggest that dentists be in no hurry to send on their money—for the way matters look now, it is not only probable that Mr. White will succeed in setting aside the Appeal Case, but also, that the Cummings patent will ultimately be overthrown. The accomplishment of so desirable a result depends however largely upon each man in the profession doing his duty, and giving aid to organizations for this purpose. H. A. S.

